# Supplementary material for: Role and mechanism of NCAPD3 in promoting malignant behaviors in gastric cancer
Source: Front Pharmacol. 2024 Apr 22;15:1341039. doi: 10.3389/fphar.2024.1341039 (PMC11070777; doi:10.3389/fphar.2024.1341039)
Supplement: Supplementary file 11 [file DataSheet2.ZIP › GSEA/GO_CC/my_analysis.Gsea.1599461408869/GO_ADHERENS_JUNCTION.html]

Details for gene set GO\_ADHERENS\_JUNCTION[GSEA]

|  || Dataset | filtered\_dataset.sample\_info.cls#WT\_versus\_NCAPD3\_MUT |
| Phenotype | sample\_info.cls#WT\_versus\_NCAPD3\_MUT |
| Upregulated in class | NCAPD3\_MUT |
| GeneSet | GO\_ADHERENS\_JUNCTION |
| Enrichment Score (ES) | -0.32787418 |
| Normalized Enrichment Score (NES) | -1.6461246 |
| Nominal p-value | 0.024324324 |
| FDR q-value | 0.11216726 |
| FWER p-Value | 0.665 |
Table: GSEA Results Summary

  

Fig 1: Enrichment plot: GO\_ADHERENS\_JUNCTION      
 Profile of the Running ES Score & Positions of GeneSet Members on the Rank Ordered List

  

| SYMBOL | TITLE | RANK IN GENE LIST | RANK METRIC SCORE | RUNNING ES | CORE ENRICHMENT || 1 | 342184 | FMN1 | 9 | 1.110 | 0.0663 | No |
| 2 | 659 | BMPR2 | 16 | 1.047 | 0.1306 | No |
| 3 | 56288 | PARD3 | 99 | 0.790 | 0.1232 | No |
| 4 | 324 | APC | 281 | 0.607 | 0.0325 | No |
| 5 | 10818 | FRS2 | 363 | 0.558 | 0.0106 | No |
| 6 | 4771 | NF2 | 428 | 0.516 | -0.0017 | No |
| 7 | 9223 | MAGI1 | 737 | 0.357 | -0.2005 | No |
| 8 | 57619 | SHROOM3 | 783 | 0.331 | -0.2113 | No |
| 9 | 1605 | DAG1 | 823 | 0.304 | -0.2195 | No |
| 10 | 55971 | BAIAP2L1 | 906 | -0.333 | -0.2568 | No |
| 11 | 301 | ANXA1 | 914 | -0.342 | -0.2394 | No |
| 12 | 9414 | TJP2 | 916 | -0.345 | -0.2175 | No |
| 13 | 55591 | VEZT | 973 | -0.389 | -0.2324 | No |
| 14 | 5818 | PVRL1 | 1073 | -0.458 | -0.2738 | No |
| 15 | 90952 | ESAM | 1149 | -0.514 | -0.2942 | Yes |
| 16 | 117178 | SSX2IP | 1163 | -0.532 | -0.2687 | Yes |
| 17 | 2043 | EPHA4 | 1228 | -0.602 | -0.2754 | Yes |
| 18 | 4301 | MLLT4 | 1237 | -0.614 | -0.2409 | Yes |
| 19 | 54800 | KLHL24 | 1287 | -0.677 | -0.2318 | Yes |
| 20 | 55691 | FRMD4A | 1295 | -0.689 | -0.1917 | Yes |
| 21 | 7414 | VCL | 1308 | -0.712 | -0.1536 | Yes |
| 22 | 10611 | PDLIM5 | 1335 | -0.758 | -0.1227 | Yes |
| 23 | 7422 | VEGFA | 1346 | -0.788 | -0.0783 | Yes |
| 24 | 10397 | NDRG1 | 1380 | -0.925 | -0.0414 | Yes |
| 25 | 23650 | TRIM29 | 1384 | -0.951 | 0.0188 | Yes |
Table: GSEA details [plain text format]

  

Fig 2: GO\_ADHERENS\_JUNCTION      
 Blue-Pink O' Gram in the Space of the Analyzed GeneSet

  

Fig 3: GO\_ADHERENS\_JUNCTION: Random ES distribution      
 Gene set null distribution of ES for **GO\_ADHERENS\_JUNCTION**
